# A maternal high-fat diet during pregnancy and lactation induced depression-like behavior in offspring and myelin-related changes in the rat prefrontal cortex

**DOI:** 10.3389/fnmol.2023.1303718

**Published:** 2024-01-03

**Authors:** Małgorzata Frankowska, Paulina Surówka, Kinga Gawlińska, Małgorzata Borczyk, Michał Korostyński, Małgorzata Filip, Irena Smaga

**Affiliations:** ^1^Department of Drug Addiction Pharmacology, Maj Institute of Pharmacology Polish Academy of Sciences, Kraków, Poland; ^2^Laboratory of Pharmacogenomics, Department of Molecular Neuropharmacology, Maj Institute of Pharmacology Polish Academy of Sciences, Kraków, Poland

**Keywords:** depression, high-fat diet, maternal diet, myelination, offspring

## Abstract

In accordance with the developmental origins of health and disease, early-life environmental exposures, such as maternal diet, can enhance the probability and gravity of health concerns in their offspring in the future. Over the past few years, compelling evidence has emerged suggesting that prenatal exposure to a maternal high-fat diet (HFD) could trigger neuropsychiatric disorders in the offspring, such as depression. The majority of brain development takes place before birth and during lactation. Nevertheless, our understanding of the impact of HFD on myelination in the offspring’s brain during both gestation and lactation remains limited. In the present study, we investigated the effects of maternal HFD (60% energy from fat) on depressive-like and myelin-related changes in adolescent and adult rat offspring. Maternal HFD increased immobility time during the forced swimming test in both adolescent and adult offspring. Correspondingly, the depressive-like phenotype in offspring correlated with dysregulation of several genes and proteins in the prefrontal cortex, especially of myelin-oligodendrocyte glycoprotein (MOG), myelin and lymphocyte protein (MAL), 2′,3′-cyclic-nucleotide 3′-phosphodiesterase (CNPase), kallikrein 6, and transferrin in male offspring, as well as of MOG and kallikrein 6 in female offspring, which persist even into adulthood. Maternal HFD also induced long-lasting adaptations manifested by the reduction of immature and mature oligodendrocytes in the prefrontal cortex in adult offspring. In summary, maternal HFD-induced changes in myelin-related genes are correlated with depressive-like behavior in adolescent offspring, which persists even to adulthood.

## 1 Introduction

In accordance with the developmental origins of health and disease, early-life environmental exposures, such as maternal diet, can enhance the probability and gravity of health concerns in their offspring in the future (Barker, 2007; Armitage et al., 2008). The preclinical studies suggest a relationship between maternal high-fat diet (HFD) and neuropsychiatric disorders including autism spectrum disorders (Gawlińska et al., 2021a,c), cognitive impairment (Ziemens et al., 2022; Smaga et al., 2023), schizophrenia (Sarker et al., 2019), depression (Giriko et al., 2013; Gawlińska et al., 2020b; Gawliński et al., 2021), and substance use disorder (Gawliński et al., 2020). Some investigators also showed that maternal HFD reduces social interactions and increases repetitive behavior (Ergaz et al., 2016; Gawlińska et al., 2020b,Gawlińska et al., 2021c), as well as evokes the disturbances in short-term memory (Smaga et al., 2023), social memory and sensorimotor gating deficits (Bordeleau et al., 2021). The behavioral changes observed during pregnancy and early childhood are linked to changes in brain structure, function, and molecular processes resulting from maternal HFD consumption (Gawlińska et al., 2021b). Additionally, our previous report showed that a maternal HFD during pregnancy and lactation provokes a depressive-like phenotype in adolescent and adult offspring (Gawlińska et al., 2020b; Gawliński et al., 2021). As the majority of brain development takes place before birth and during lactation, maternal nutrition has been recognized as a crucial factor for brain growth and maturation (Gawlińska et al., 2020a). Nevertheless, our understanding of the impact of HFD during both gestation and lactation on offspring brain myelination remains limited.

Oligodendrocytes, responsible for forming myelin layers around neuronal axons, exhibit high sensitivity to changes in local homeostasis (Smaga, 2022). Hence, its survival and/or maturation may be constrained and halted due to various types of pathological signals, including environmental factors such as maternal diet (Smaga, 2022). Throughout development, oligodendrocytes undergo a multi-stage maturation process to acquire the ability for myelination. Oligodendrocyte maturation is marked by several concurrent indicators, while their capacity for myelination is associated with the activation of specific genes encoding proteins and lipids, as well as the formation of multiple lipid-rich layers within tightly packed membranes (Janowska and Sypecka, 2018). Myelin is a well-structured and densely layered substance that serves to insulate nerve cell axons, facilitating efficient signal conduction. Additionally, it plays several essential roles within the nervous system, including shielding axons to preserve electrical signals, promoting axonal growth, regulating metabolism, ensuring integrity and survival (Brady et al., 1999; Fünfschilling et al., 2012), as well as contributing to the regulation of neurotransmission (Jang et al., 2019), neuronal circuits (Monje, 2018), and synaptic plasticity (Zemmar et al., 2018).

In the mature brain, a substantial pool of oligodendrocyte progenitor cells remains even after myelination, and these cells express a diverse range of neurotransmitter and neuroactive ligand receptors (Janowska and Sypecka, 2018). Oligodendrocyte progenitor cells actively influence interactions with neurons, playing a prominent role in brain neuronal activity (Thornton and Hughes, 2020). This influence is mediated through glutamatergic and γ-aminobutyric acid (GABA)-ergic neurons that regulate their proliferation. Additionally, these cells enhance the frequency and amplitude of spontaneous glutamatergic inputs during the initial three postnatal weeks (Mangin et al., 2008). Moreover, α-amino-3-hydroxy-5-methyl-4-isoxazolepropionic acid (AMPA) receptor-dependent signaling is implicated in the promotion of oligodendrocyte development and myelination during postnatal development (Thornton and Hughes, 2020) and provides membrane depolarization and local calcium influx (Maldonado and Angulo, 2015). It appears that myelin and oligodendrocytes, through their distinct impacts on cellular processes, may serve as pivotal elements connecting previously suggested theories of depression and could play a substantial role in the development of this condition. Recent observations highlighted the potential role of myelin alteration in depression in preclinical and clinical studies (Smaga, 2022), while 5 weeks of HFD before mating in female mice provoked decreases in myelination in the medial cortex in male offspring (Graf et al., 2016).

Based on the crosstalk among maternal diet, myelination, and depression, we decided to perform the effect of maternal HFD during pregnancy and lactation on the depressive-like phenotype in adolescent [at postnatal day (PND) 28] and adult (at PND 63) offspring. Next, we addressed maternal HFD programs on myelination processes (mRNA, protein levels, and immunofluorescence staining) in the brain structures involved in the pathogenesis of depression - prefrontal cortex (PFCTX) and hippocampus - in adolescent rats whose mothers fed HFD. Finally, we investigated if impaired myelination might persist even to adulthood. The PFCTX and hippocampus are integral to the regulation of emotional behaviors and cognitive functions, with the PFCTX playing a pivotal role in executive functions and the hippocampus being central to memory and stress responses (McEwen and Morrison, 2013). Both regions are particularly sensitive to environmental influences during development, including dietary factors. These areas are not only interconnected but also exhibit late maturation and distinct myelination patterns that continue to evolve into early adulthood.

## 2 Materials and methods

### 2.1 Behavioral experiments

#### 2.1.1 Animals and diets

**FIGURE 1:**
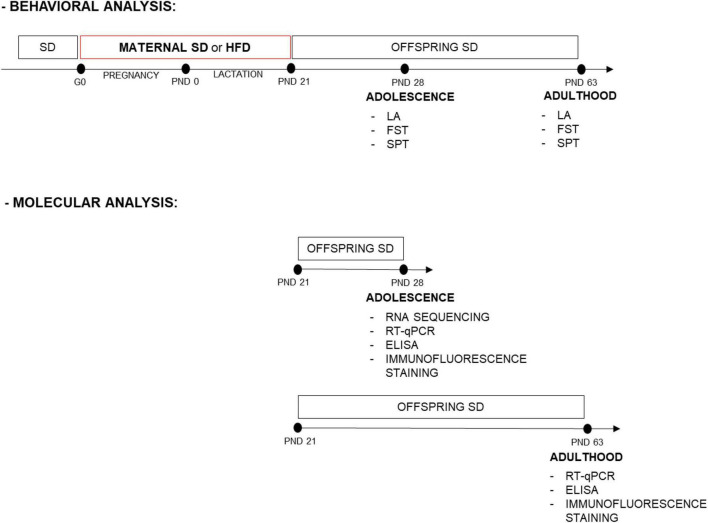
Experimental design and timeline. Dams were fed a standard diet (SD) or high-fat diet (HFD) during pregnancy and lactation [from gestational day (G) 0 to postnatal day (PND) 21]. Adolescent and adult male and female offspring were assessed for behavioral tests. Separate sets of animals, at PND 28 the prefrontal cortex (PFCTX) and hippocampus were isolated for gene expression profiling, mRNA, and protein level analysis, while at PND 63 mRNA and protein levels were analyzed in the PFCTX. Adolescent and adult offspring were also assessed for immunofluorescence staining. FST, forced swimming test; LA, locomotor activity; SPT, sucrose preference test.

Wistar Han rats sourced from Charles River, Sulzfeld, Germany, were housed in conventional plastic rodent cages maintained at a room temperature of 22 ± 2°C and a relative humidity of 55 ± 10%. They were subjected to a 12-h light-dark cycle, with lights on at 6:00 a.m., and provided unrestricted access to both food and water. Following an acclimatization period, female rats (200–240 g) were paired with male counterparts during the proestrus phase for mating. The gestation was confirmed by examining vaginal smears for the presence of sperm. Dams were individually housed and randomly assigned to two groups: standard diet (SD, 10% energy from fat, 3.51 kcal/g; C1090-10, Altromin, Lage, Germany) or HFD (60% energy from fat, 5.23 kcal/g; C1090-60, Altromin, Lage, Germany), and they all had free access to the diets during pregnancy (21 days) and lactation (21 days). After weaning, offspring at PND 22 were separated according to sex, housed 3–4 per cage, and switched to an SD. Male and female offspring were used in the present study. The experimental design and timeline are presented in Figure 1. The present study was carried out under the European Union Directive 2010/63/EU and with approval from the Local Ethics Commission at the Maj Institute of Pharmacology Polish Academy of Sciences, Kraków, Poland (255/2021, 26 August 2021).

#### 2.1.2 Locomotor activity

Locomotor activity was recorded individually for each animal twice at PNDs 28 and 63 (*n* = 10 rats/group) in Opto-Varimex cages (Columbus Instruments, Columbus, USA) linked online to an IBM-compatible PC. Each cage, measuring 43 cm × 44 cm, was outfitted with 15 infrared emitters positioned along both the x and y axes, situated 3 cm above the floor. Correspondingly, an equal number of receivers were placed on the opposite walls of the cage. The analysis of the rats’ behavior was conducted using Auto-Track software (Columbus Instruments, Columbus, USA). Locomotor activity was defined as the interruption of three consecutive photo beams, representing horizontal movement, and was quantified as the distance traveled in centimeters during a 5-min trial.

#### 2.1.3 Forced swimming test

On the 1st day of the forced swim (i.e., pre-test), one cohort of rats (*n* = 10 rats/group) at PND 27 were placed individually in a cylinder (50 cm high × 23 cm in diameter) filled to a 30-cm depth with water (25 ± 1°C) for 15 min. Subsequently, they were gently removed from the water, dried using towels, and provided with warmth in a designated enclosure for 15 min. Afterward, the rats were returned to their respective home cages, following the procedure described previously (Smaga et al., 2012). The cylinders were emptied and cleaned between rats. Next, rats at PNDs 28 and 63 were tested for 5 min (300 s) under identical conditions, and the immobility, swimming, and climbing time were measured, recorded by a digital camera and then analyzed.

#### 2.1.4 Sucrose preference test

Two separate cohorts of adolescent (*n* = 8 rats/group) and adult (*n* = 8 rats/group) rats were individually placed in cages with free access to food and water. The water bottle’s placement was altered, shifting it from the left side to the right. Following this change, the animals were allowed access to two water bottles for a duration of 4 days, and their water intake was carefully monitored over the course of 2 days. Subsequently, the animals were provided with two premeasured bottles—one containing tap water and the other a 1% sucrose solution—for a 48-h period. The bottles were reweighed after the first 24 h, and then their positions were interchanged. After another 48 h, the sucrose solution was substituted with plain water, and the measurement of water consumption continued. Sucrose and water consumption were calculated as milliliters of sucrose solution (or water) consumed per kilogram of animal body weight per day (ml/kg/day). Sucrose preference was calculated according to the formula: % Preference = [(sucrose intake/total intake: sucrose + water) × 100].

### 2.2 Brain structure isolation

Separate cohorts of animals were used for molecular analyses. PFCTX and hippocampus were isolated according to the Rat Brain Atlas (Paxinos and Watson, 1998) in rat offspring at PND 28 (*n* = 10 rats/group) or PND 63 (*n* = 8–10 rats/group).

### 2.3 Molecular analyses

#### 2.3.1 RNA isolation

RNA was extracted in accordance with the manufacturer’s guidelines and subsequently subjected to additional purification using the RNA Mini Kit (A&A Biotechnology, Gdańsk, Poland). The total RNA concentration was measured using an ND-1000 Spectrometer (NanoDrop Technologies Inc., Wilmington, DE, USA).

#### 2.3.2 RNA sequencing

RNA quality assessment was conducted using an RNA 6000 Nano LabChip Kit and an Agilent Bioanalyzer 2100 (Agilent, USA). Based on the RNA integrity number (RIN > 7.5) values, samples from 10 animals at PND 28 from each group were chosen for RNA sequencing (PFCTX and hippocampus; 80 samples total). RNA sequencing was performed as an external service by Novogene. For each sample, precisely 1 μg of RNA was utilized as the starting material for RNA sample preparation. To enhance the concentration of mRNA originating from eukaryotic organisms, oligo(dT) beads were employed. Following this, sequencing libraries were meticulously crafted in accordance with the manufacturer’s instructions using the NEBNext Ultra II Directional RNA Library Prep Kit designed for Illumina by NEB in the USA. These libraries comprised comprehensive cDNA fragments, each spanning a length range of 250 to 300 bp, which were then subjected to sequencing on an Illumina system (PE150, 20 M reads per sample). The raw reads were submitted to Sequence Read Archive (SRA) under the PRJNA977708 BioProject.

#### 2.3.3 Bioinformatic analyses

RNA reads were processed with an automated Intelliseq-flow RNAseq pipeline.^1^ Within this pipeline, raw reads were mapped to the Rnor_6.0 reference genome from Ensembl (version 104) using the STAR tool (version 2.7.3a). The gene expression level was determined using the feature Counts tool (version 2.0.0). GTF file from the Ensembl database (version 104) was used as a reference. Read quality analysis was performed with the FastQC tool (version FastQC-v0.11.9) and the MultiQC tool (version 1.10.1) was used to visualize QC results. Data analysis was performed with R 4.2.1 and the code is available in the project’s repository.^2^ Functional enrichment analysis was performed for top genes regulated by the diet factor (FDR diet < 0.01, *n* = 98 genes) with the Tabula Muris database using the Enrichr tool (Xie et al., 2021). Data was analyzed with the Edger library, a gene was considered regulated if it passed the FDR threshold of 0.1 (10%). The differential expression of 41 pre-selected genes was analyzed.

#### 2.3.4 RT-qPCR analyses

The synthesis of cDNA through reverse transcription, utilizing equal quantities of RNA, was carried out employing the High-Capacity cDNA Reverse Transcription Kit (Thermo Fisher Scientific, Life Technologies, Waltham, MA, USA). RT-qPCR was performed by using the QuantStudio 3 (Applied Biosystems, Foster City, CA, USA) and TaqMan Gene Expression Assays (Applied Biosystems, Waltham, MA, USA), i.e., *Mog* (Rn00575354_m1), *Mal* (Rn00562993_m1), *Mobp* (Rn00824538_m1), *Mag* (Rn01457782_m1), *Cnp* (Rn01399463_m1), *Gjb1* (Rn01641031_s1), *Cldn11* (Rn00584941_m1), *Klk6* (Rn00569838_m1), *Pllp* (Rn00574751_g1), *Tf* (Rn01445482_m1), *Tfrc* (Rn01474701_m1). The PCR cycling conditions were as follows: an initial step at 95°C for 10 min followed by 40 cycles at 95°C for 15 s and then 60°C for 60 s. The relative level of mRNA was assessed using the comparative CT method (2^–ΔΔCt^) and normalized to the level of the eukaryotic 18S ribosomal RNA (Hs99999901_s1). The values are expressed as the fold change relative to the control (SD group).

#### 2.3.5 ELISAs

Significant changes in the mRNA level were validated at the protein level using Rat ELISA Kits (Bioassay Technology Laboratory, Shanghai, China), i.e., myelin-oligodendrocyte glycoprotein (MOG) (E0859Ra), myelin and lymphocyte protein (MAL) (E3379Ra), myelin-associated oligodendrocyte basic protein (MOBP) (E3390Ra), myelin-associated glycoprotein (MAG) (E1102Ra), 2′,3′-cyclic-nucleotide 3′-phosphodiesterase (CNPase) (E3377Ra), Gap junction beta-1 protein (GJB1) (E3378Ra), Claudin 11 (E3331Ra), Kallikrein 6 (E1133Ra), transferrin (E1636Ra), and transferrin receptor (E0782Ra) following manufacturers’ protocols. Briefly, frozen brain structures were rapidly homogenized in cold PBS (pH 7.4) containing a mixture of protease and phosphatase inhibitors (Sigma-Aldrich, St. Louis, MO, USA). This homogenization was achieved using a Bioprep-24 homogenizer ball (Aosheng, Hangzhou, China) running for 10 s at 10,000 rpm. Subsequently, the homogenates were subjected to centrifugation at 5,000 × *g* for 5 min, and the resulting supernatants were promptly collected. The total protein concentration in these supernatants was determined using a bicinchoninic acid (BCA) protein assay kit (Serva, Heidelberg, Germany). Duplicate samples and a series of standards were then transferred onto enzyme-linked immunosorbent assay (ELISA) plates. The absorbance was measured at a wavelength of λ = 450 nm using a Multiskan Spectrum spectrophotometer (Thermo LabSystems, Philadelphia, PA, USA). The concentration of proteins was calculated from standard curves and expressed as ng/mg of protein.

#### 2.3.6 Confocal microscopy

At 28 (*n* = 6 rats/group) and 63 PND (*n* = 5–7 rats/group), the rats were anesthetized with pentobarbital and subsequently underwent intracardial perfusion with a solution consisting of 4% formaldehyde dissolved in 100 mM PBS with a pH of 7.4. Following perfusion, the brains were carefully removed and immersed in the same fixative for a duration of 12 h. To facilitate tissue preparation, the brain tissue was subjected to a permeation process using a 10% w/v sucrose solution for a period of 7 days. After this initial step, it was transitioned to a 30% w/v sucrose solution in PBS and maintained at a temperature range of 4–8°C for at least 48 h. Subsequently, the brains were fully frozen by placing them on dry ice, then sliced into 16-μm coronal sections using a cryostat (Leica Microsystems, Nussloch, Germany). These sections were stored at a temperature of −20°C until they were ready for further analysis. The rat brain sections were incubated in 4% formaldehyde for 15 min, rinsed with PBS (pH = 7.4), and subjected to a permeabilization procedure for 30 min in PBS with 0.5% Triton X-100. Next, the sections were incubated for 30 min with 5% bovine serum, and subsequently incubated overnight at 4°C with primary antibodies. The following antibodies were used in this study: rabbit anti-NG2 (1:50; AB5320, Merck), mouse anti-CC-1 (1:50; ab16794, Abcam), and goat anti-Olig2 (1:50; AF2418-SP, Bio-techne). After washing with PBS containing 0.1% Tween 20, the sections were incubated in the dark for 1 h with corresponding secondary antibodies (1:500; Life Technologies): donkey anti-mouse Alexa Fluor 488 (A-21202), donkey anti-rabbit Alexa Fluor 647 (A-31573), and donkey anti-goat Alexa Fluor 568 (A-11057). Then, sections were embedded in a mounting medium with 4’,6-diamidino-2-phenylindole (DAPI) (F6057, Sigma-Aldrich, St. Louis, MO, USA) and sealed with cover glass. Finally, the images from PFCTX were captured by a scanning confocal microscope (Leica SP8 WLL) at 20 × magnification of HC PL APO 20x NA = 0.75 CS2 objective. The FIJI open-source software or LASX was used to quantification of the number of positive stained cells in the sections. The number of positive stained cells was counted in the prelimbic, infralimbic, and cingulate cortex in 2- 4 random squares (1,000 × 1,000 pixels) and then averaged. The values are expressed as the % of the control (SD group).

### 2.4 Statistical analyses

The GraphPad Prism 10 (GraphPad, La Jolla, CA, USA) was used to perform analyses. For each parameter, the normality of the distribution was assessed using the Shapiro–Wilk test and after checking all the assumptions (i.e., normal distribution, equality of variance), the appropriate statistical tests were applied. Statistical analyses of behavioral effects were performed using two-way analyses of variance (ANOVA) with Sidak’s test, while for molecular analysis the Student’s *t*-test was used. The data are presented as the mean ± SEM. *P* < 0.05 was considered statistically significant.

## 3 Results

### 3.1 Behavioral experiments

#### 3.1.1 Locomotor activity

The maternal HFD did not change the spontaneous locomotor activity in adolescent [*interaction F*(1,36) = 0.003, *p* = 0.954; *sex F*(1,36) = 3.119, *p* = 0.086; *diet F*(1,36) = 1.512, *p* = 0.227] and adult offspring [*interaction F*(1,36) = 0.1, *p* = 0.754; *sex F*(1,36) = 0.218, *p* = 0.644; *diet F*(1,36) = 0.167, *p* = 0.685] (Table 1).

**TABLE 1:** Effects of maternal high-fat diet (HFD) during pregnancy and lactation on the locomotor activity of adolescent and adult offspring.

Age	Sex	SD [distance traveled (cm)]	HFD [distance traveled (cm)]
Adolescent	Male	1,484 ± 117.9	1,351 ± 122.7
	Female	1,290 ± 125.4	1,144 ± 82.1
Adult	Male	1,060 ± 54.4	1,104 ± 86.8
	Female	1,108 ± 34.9	1,114 ± 59.2

HFD, high-fat diet; SD, standard diet. Data are presented as the mean ± SEM. *N* = 10 rats/group.

#### 3.1.2 Forced swimming test

**FIGURE 2:**
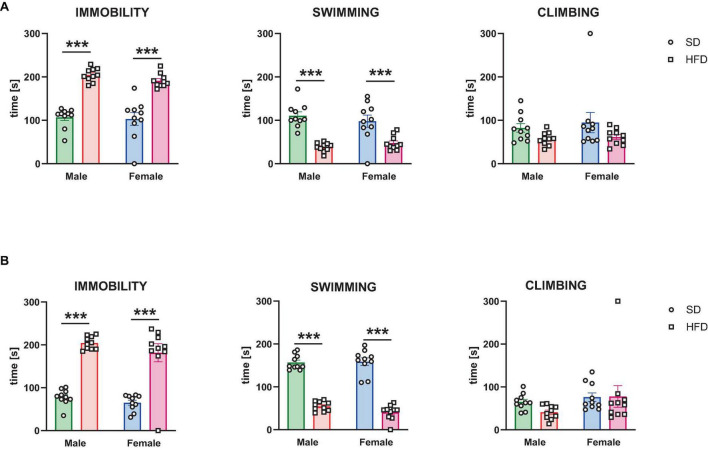
The effects of maternal high-fat diet (HFD) during pregnancy and lactation on depressive-like behavior were examined in the forced swimming test in **(A)** adolescent; and **(B)** adult offspring (male and female). Data of immobility, swimming, and climbing time in the forced swimming test are presented as the mean ± SEM with individual value plots. *N* = 10 rats/group. ***p < 0.001 vs. standard diet (SD).

The effects of modified maternal HFD on depressive-like behavior in the male and female offspring evaluated in the forced swimming test are shown in Figure 2. In adolescent offspring two-way ANOVA indicated a significant effect of diet on time of immobility [*interaction F*(1,36) = 0.3, *p* = 0.587; *sex F*(1,36) = 0.954, *p* = 0.335; *diet F*(1,36) = 109.9, *p* < 0.0001], swimming [*interaction F*(1,36) = 1.544, *p* = 0.222; *sex F*(1,36) = 0.035, *p* = 0.853; *diet F*(1,36) = 48.79, *p* < 0.0001], and climbing [*interaction F*(1,36) = 0.096, *p* = 0.759; *sex F*(1,36) = 0.392, *p* = 0.535; *diet F*(1,36) = 4.885, *p* = 0.034] (Figure 2A). *Post hoc* analyses revealed that adolescent male and female offspring following a maternal HFD showed a significantly increased time of immobility (*p* < 0.001) and decreased time of swimming (*p* < 0.001) compared to their corresponding SD control group (Figure 2A).

In adult offspring two-way ANOVA indicated a significant effect of diet on time of immobility [*interaction F*(1,36) = 0.17, *p* = 0.682; *sex F*(1,36) = 2.346, *p* = 0.134; *diet F*(1,36) = 109.3, *p* < 0.0001], swimming [*interaction F*(1,36) = 1.711, *p* = 0.199; *sex F*(1,36) = 1.002, *p* = 0.323; *diet F*(1,36) = 316.7, *p* < 0.0001], but not on time of climbing [*interaction F*(1,36) = 0.833, *p* = 0.368; *sex F*(1,36) = 2.882, *p* = 0.098; *diet F*(1,36) = 0.639, *p* = 0.429] (Figure 2B). *Post hoc* analyses revealed that adult male and female offspring following a maternal HFD showed a significantly increased time of immobility (*p* < 0.001) and decreased time of swimming (*p* < 0.001) compared to their corresponding SD control group (Figure 2B).

#### 3.1.3 Sucrose preference test

A two-way ANOVA showed that exposure to modified maternal diet did not alter sucrose preference in adolescent offspring [*interaction F*(1,28) = 2.809, *p* = 0.105; *sex F* (1,28) = 0.099, *p* = 0.755; *diet F*(1,28) = 0.252, *p* = 0.62], however, a maternal HFD changed sucrose preference in adulthood [*interaction F*(1,28) = 1.471, *p* = 0.235; *sex F*(1,28) = 1.13, *p* = 0.297; *diet F*(1,28) = 12.7, *p* = 0.001] (Table 2). *Post hoc* analyses showed that maternal HFD significantly decreased the preference for natural reward (sucrose) in adult females (*p* < 0.01) (Table 2).

**TABLE 2:** Effects of maternal high-fat diet (HFD) during pregnancy and lactation on the sucrose consumption in preference test of adolescent and adult offspring.

Age	Sex	SD [sucrose preference (%)]	HFD [sucrose preference (%)]
Adolescent	Male	91.79 ± 1.5	88.47 ± 1.3
	Female	86.16 ± 5.2	92.32 ± 0.9
Adult	Male	96.86 ± 0.5	93.11 ± 1.1
	Female	97.10 ± 0.4	82.57 ± 2.9**

HFD, high-fat diet; SD, standard diet. Data are presented as the mean ± SEM. *N* = 8 rats/group.

**p < 0.01 vs. corresponding SD control. Mean values that are highlighted in bold indicate statistical significance.

### 3.2 Molecular analyses

#### 3.2.1 Adolescent offspring

##### 3.2.1.1 RNA sequencing

**FIGURE 3:**
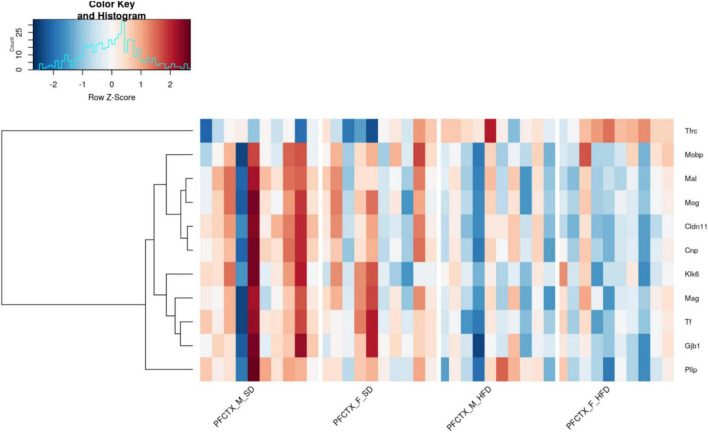
Effect of a maternal high-fat diet (HFD) during pregnancy and lactation on 11 top selected gene expression using RNA sequencing in the prefrontal cortex (PFCTX) of adolescent male (M) and female (F) offspring (FDR < 0.1). Gene transcript abundance is represented as a z-score, red colors indicate higher levels and blue colors - lower levels. SD, standard diet.

Based on a general RNAseq analysis 3310 genes were regulated by maternal HFD (FDR < 10%). Enrichment analysis of the top differentially-expressed genes with the Tabula Muris dataset (Tabula Muris Consortium et al., 2018) showed significant enrichment only for genes expressed in oligodendrocytes (adjusted *p*-value < 0.0001, Odds Ratio 15.57) (Supplementary Table 1). Further, we focused our analyses on regulated transcripts from the pre-selected list of genes related to myelination. Non-parametric statistical analysis revealed that 37 genes related to myelination were differentially expressed between the hippocampus and PFCTX of adolescent offspring, 13 genes were regulated by sex, and 11 genes were regulated by maternal diet in the PFCTX (Figure 3). None of the selected genes were significantly regulated in the hippocampus by a maternal HFD (for full results see Supplementary Table 2).

##### 3.2.1.2 mRNA levels

**FIGURE 4:**
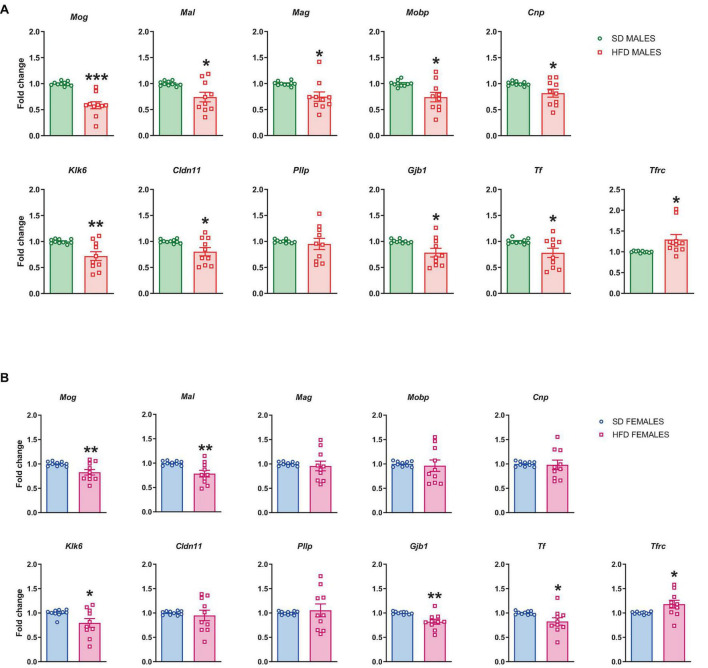
Changes in the mRNA levels in the prefrontal cortex (PFCTX) of adolescent **(A)** male and **(B)** female offspring whose mothers were fed on a standard diet (SD) or high-fat diet (HFD) during pregnancy and lactation. Data are presented as the mean ± SEM with individual value plots. *N* = 10 rats/group. *p < 0.05, **p < 0.01, ***p < 0.001 vs. SD.

All significant maternal diet-induced changes related to myelination seen in RNA sequencing analysis were validated at mRNA levels. RT-qPCR confirmed the reduced mRNA levels of *Mog* (*t* = 6.073, df = 18, *p* < 0.0001), *Mal* (*t* = 2.806, df = 18, *p* = 0.012), *Mag* (*t* = 2.77, df = 18, *p* = 0.013), *Mobp* (*t* = 2.838, df = 18, *p* = 0.011), *Cnp* (*t* = 2.385, df = 18, *p* = 0.028), *Klk6* (*t* = 3.267, df = 18, *p* = 0.004), *Cldn11* (*t* = 2.467, df = 18, *p* = 0.024), *Gjb1* (*t* = 2.574, df = 18, *p* = 0.019), and *Tf* (*t* = 2.425, df = 18, *p* = 0.026), and the increased the mRNA level of *Tfrc* (*t* = 2.44, df = 18, *p* = 0.025) in the PFCTX of adolescent male offspring following a maternal HFD in pregnancy and lactation, but not mRNA level of *Pllp* (*t* = 0.461, df = 18, *p* = 0.651) (Figure 4A).

At the same time, the reduced mRNA levels of *Mog* (*t* = 2.909, df = 18, *p* = 0.009), *Mal* (*t* = 3.111, df = 18, *p* = 0.006), *Klk6* (*t* = 2.202, df = 18, *p* = 0.041), *Gjb1* (*t* = 3.473, df = 18, *p* = 0.003), and *Tf* (*t* = 2.264, df = 18, *p* = 0.036), and the increased the mRNA level of *Tfrc* (*t* = 2.256, df = 18, *p* = 0.037) were observed in the PFCTX of adolescent female offspring following a maternal HFD in pregnancy and lactation (Figure 4B). Interestingly, mRNA levels of *Mag* (*t* = 0.428, df = 18, *p* = 0.674), *Mobp* (*t* = 0.316, df = 18, *p* = 0.756), *Cnp* (*t* = 0.19, df = 18, *p* = 0.851), *Cldn11* (*t* = 0.465, df = 18, *p* = 0.647), and *Pllp* (*t* = 0.409, df = 18, *p* = 0.687) did not change in the PFCTX of female offspring whose mothers were fed the HFD (Figure 4B).

##### 3.2.1.3 Protein levels

After observing significant differences in mRNA levels, these differences were validated at the protein level. Statistically significantly lower levels of the MOG (*t* = 2.182, df = 18, *p* = 0.043), MAL (*t* = 2.207, df = 18, *p* = 0.041), MAG (*t* = 2.58, df = 18, *p* = 0.019), MOBP (*t* = 2.223, df = 18, *p* = 0.039), CNPase (*t* = 2.133, df = 18, *p* = 0.047), kallikrein 6 (*t* = 2.425, df = 18, *p* = 0.026), claudine 11 (*t* = 4.207, df = 18, *p* = 0.001), GJB1 (*t* = 2.612, df = 18, *p* = 0.018), and transferrin (*t* = 2.124, df = 18, *p* = 0.048) expression levels were detected in the PFCTX in adolescent male offspring whose mothers were fed an HFD during pregnancy and lactation, while the protein expression level of transferrin receptor did not change (*t* = 1.538, df = 18, *p* = 0.141) in this structure (Table 3).

**TABLE 3:** Effects of maternal high-fat diet (HFD) during pregnancy and lactation on the protein expression levels in the prefrontal cortex (PFCTX) of adolescent and adult offspring.

Sex	Protein	SD (ng/mg protein)	HFD (ng/mg protein)
**Adolescent offspring**			
Male	MOG	3.13 ± 0.16	2.61 ± 0.18*
	MAL	7.15 ± 0.28	6.06 ± 0.41*
	MAG	4.13 ± 0.17	3.37 ± 0.24*
	MOBP	3.77 ± 0.10	3.13 ± 0.27*
	CNPase	4.01 ± 0.19	3.36 ± 0.24*
	Kallikrein 6	8.16 ± 0.29	6.68 ± 0.53*
	Claudine 11	5.42 ± 0.32	3.70 ± 0.26***
	GJB1	5.19 ± 0.19	4.26 ± 0.30*
	Transferrin	0.73 ± 0.02	0.63 ± 0.04*
	Transferrin receptor	25.31 ± 1.37	21.79 ± 1.84
Female	MOG	3.01 ± 0.14	3.15 ± 0.11
	MAL	3.45 ± 0.16	4.05 ± 0.16
	Kallikrein 6	6.83 ± 0.30	7.32 ± 0.28
	GJB1	4.78 ± 0.14	4.83 ± 0.17
	Transferrin	0.68 ± 0.02	0.68 ± 0.03
	Transferrin receptor	20.96 ± 0.91	22.00 ± 1.14
**Adult offspring**			
Male	MOG	3.76 ± 0.17	3.12 ± 0.10**
	MAL	8.54 ± 0.35	7.32 ± 0.25*
	CNPase	4.00 ± 0.24	3.16 ± 0.15*
	Kallikrein 6	7.46 ± 0.47	6.23 ± 0.29*
	Transferrin	0.88 ± 0.03	0.77 ± 0.02*
Female	MOG	3.29 ± 0.13	3.00 ± 0.03*
	MAL	7.09 ± 0.44	7.57 ± 0.22
	Kallikrein 6	7.01 ± 0.31	6.11 ± 0.16*
	Transferrin	0.75 ± 0.03	0.80 ± 0.03
	Transferrin receptor	20.14 ± 0.68	19.36 ± 1.27

CNPase, 2′,3′-cyclic-nucleotide 3′-phosphodiesterase; GJB1, gap junction beta-1 protein; HFD, high-fat diet; MAG, myelin associated glycoprotein; MAL, myelin and lymphocyte protein; MOBP, myelin-associated oligodendrocyte basic protein; MOG, myelin-oligodendrocyte glycoprotein; SD, standard diet. Data are presented as the mean ± SEM. *N* = 8–10 rats/group.

*p < 0.05,

**p < 0.01,

***p < 0.001 vs. corresponding SD control. Mean values that are highlighted in bold indicate statistical significance.

Surprisingly, no significant differences in the MOG (*t* = 0.765, df = 18, *p* = 0.455), MAL (*t* = 0.011, df = 18, *p* = 0.992), kallikrein 6 (*t* = 1.215, df = 18, *p* = 0.24), GJB1 (*t* = 0.207, df = 18, *p* = 0.838), transferrin (*t* = 0.029, df = 18, *p* = 0.978), and transferrin receptor (*t* = 0.714, df = 18, *p* = 0.484) expression levels were observed in adolescent female offspring following a maternal HFD (Table 3).

##### 3.2.1.4 Quantification of oligodendrocyte precursor cells and mature oligodendrocyte cells

**FIGURE 5:**
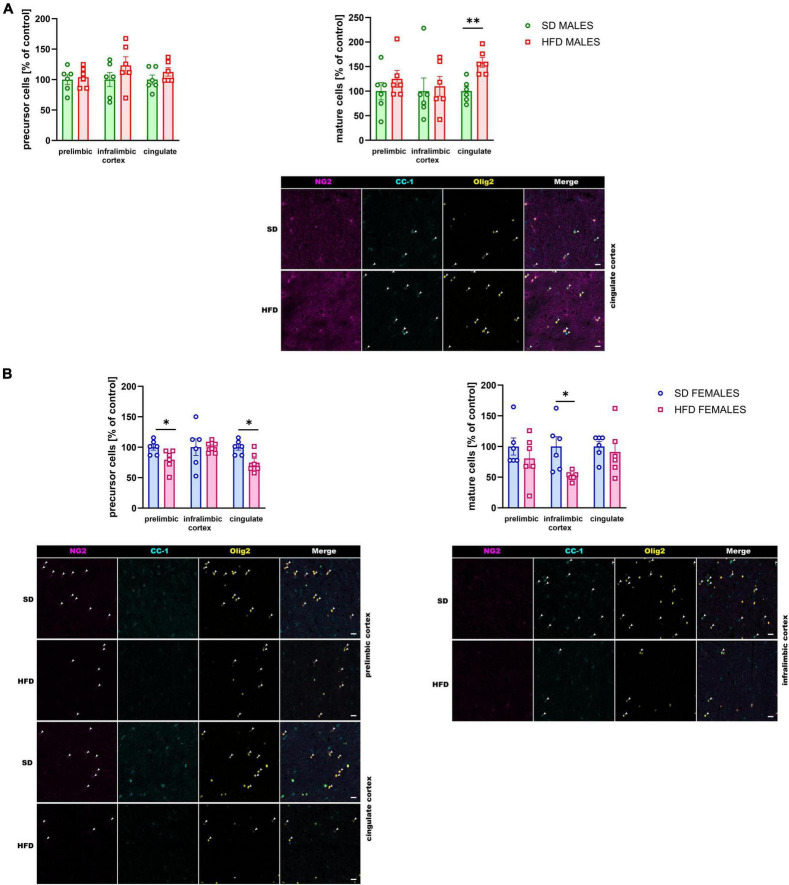
Effect of a maternal high-fat diet (HFD) during pregnancy and lactation on the number of oligodendrocyte precursor cells (NG2^+^/Olig2^+^ cells) and mature oligodendrocyte cells (CC-1^+^/Olig2^+^ cells) in the prelimbic, infralimbic, and cingulate cortex of adolescent **(A)** male and **(B)** female offspring. Representative images of channels showing NG2, CC-1, Olig2, and Merge immunofluorescence staining were shown. The scale bar represents 20 μm. Data are presented as the mean ± SEM. *N* = 5–7 rats/group. *p < 0.05, **p < 0.01 vs. standard diet (SD).

The direct consequences of maternal diet on myelination impairment were evaluated using immunofluorescence staining for visualization of specific markers of oligodendrocyte lineage cells. A maternal HFD during pregnancy and lactation increased the number of mature oligodendrocyte cells (*t* = 4.360, df = 10, *p* = 0.001) only in the cingulate cortex of adolescent male rats, without the effect on the number of oligodendrocyte precursor cells (Figure 5A).

**FIGURE 6:**
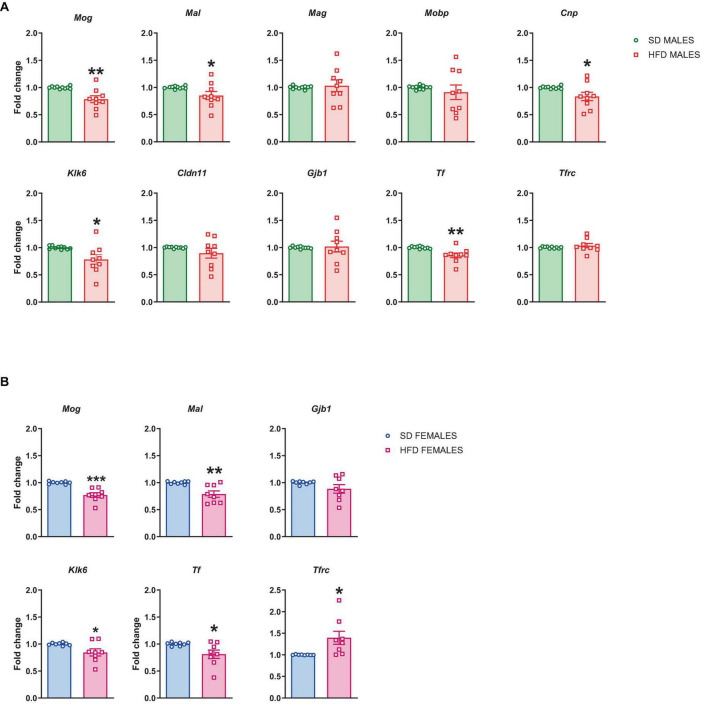
Changes in the mRNA levels in the prefrontal cortex (PFCTX) of adult **(A)** male and **(B)** female offspring whose mothers were fed on a standard diet (SD) or high-fat diet (HFD) during pregnancy and lactation. Data are presented as the mean ± SEM with individual value plots. *N* = 8–10 rats/group. *p < 0.05, **p < 0.01, ***p < 0.001 vs. SD.

At the same time, the reduction of the number of oligodendrocyte precursor cells was observed in the prelimbic (*t* = 2.490, df = 10, *p* = 0.032) and cingulate (*t* = 3.103, df = 10, *p* = 0.011) cortex of adolescent female offspring whose mothers were fed HFD during pregnancy and lactation compared to SD (Figure 6B). Interestingly, the reduction of mature oligodendrocytes were shown in the infralimbic (*t* = 2.965, df = 10, *p* = 0.014) cortex of the adolescent female rats following maternal HFD (Figure 5B).

#### 3.2.2 Adult offspring

##### 3.2.2.1 mRNA levels

Furthermore, we checked if the above changes in the mRNA level persist to adulthood. The reduced mRNA levels of *Mog* (*t* = 3.591, df = 17, *p* = 0.002), *Mal* (*t* = 2.119, df = 17, *p* = 0.049), *Cnp* (*t* = 2.245, df = 17, *p* = 0.038), *Klk6* (*t* = 2.581, df = 17, *p* = 0.019), and *Tf* (*t* = 3.481, df = 17, *p* = 0.003) were also observed in adulthood in male offspring following maternal HFD during pregnancy and lactation, while the mRNA levels of *Mag* (*t* = 0.304, df = 17, *p* = 0.765), *Mobp* (*t* = 0.699, df = 17, *p* = 0.494), *Cldn11* (*t* = 1.236, df = 17, *p* = 0.233), *Gjb1* (*t* = 0.189, df = 17, *p* = 0.852), and *Tfrc* (*t* = 0.894, df = 17, *p* = 0.384) did not change in the PFCTX of male adult offspring (Figure 6A).

In female adult offspring the reduced mRNA levels of *Mog* (*t* = 5.215, df = 14, *p* = 0.0001), *Mal* (*t* = 3.574, df = 14, *p* = 0.003), *Klk6* (*t* = 2.332, df = 14, *p* = 0.035), and *Tf* (*t* = 2.402, df = 14, *p* = 0.031), and the increased the mRNA level of *Tfrc* (*t* = 2.622, df = 14, *p* = 0.02) were observed, while the mRNA level of *Gjb1* (*t* = 1.447, df = 14, *p* = 0.17) was not altered in the PFCTX of female adult offspring whose mothers were fed the HFD compared to SD control (Figure 6B).

##### 3.2.2.2 Protein levels

A maternal HFD during pregnancy and lactation provoked a reduction in the MOG (*t* = 3.207, df = 14, *p* = 0.006), MAL (*t* = 2.815, df = 14, *p* = 0.014), CNPase (*t* = 2.967, df = 14, *p* = 0.01), kallikrein 6 (*t* = 2.249, df = 14, *p* = 0.041), and transferrin (*t* = 2.928, df = 14, *p* = 0.011) expression levels in the PFCTX in adult male offspring. At the same time, the reduction in the MOG (*t* = 2.159, df = 14, *p* = 0.049) and kallikrein 6 (*t* = 2.586, df = 14, *p* = 0.022) expression levels, but not in the MAL (*t* = 0.96, df = 14, *p* = 0.354), transferrin (*t* = 1.142, df = 14, *p* = 0.273) and transferrin receptor (*t* = 0.536, df = 14, *p* = 0.6) expression levels, were observed in the PFCTX of adult female offspring (Table 3).

##### 3.2.2.3 Quantification of oligodendrocyte precursor cells and mature oligodendrocyte cells

**FIGURE 7:**
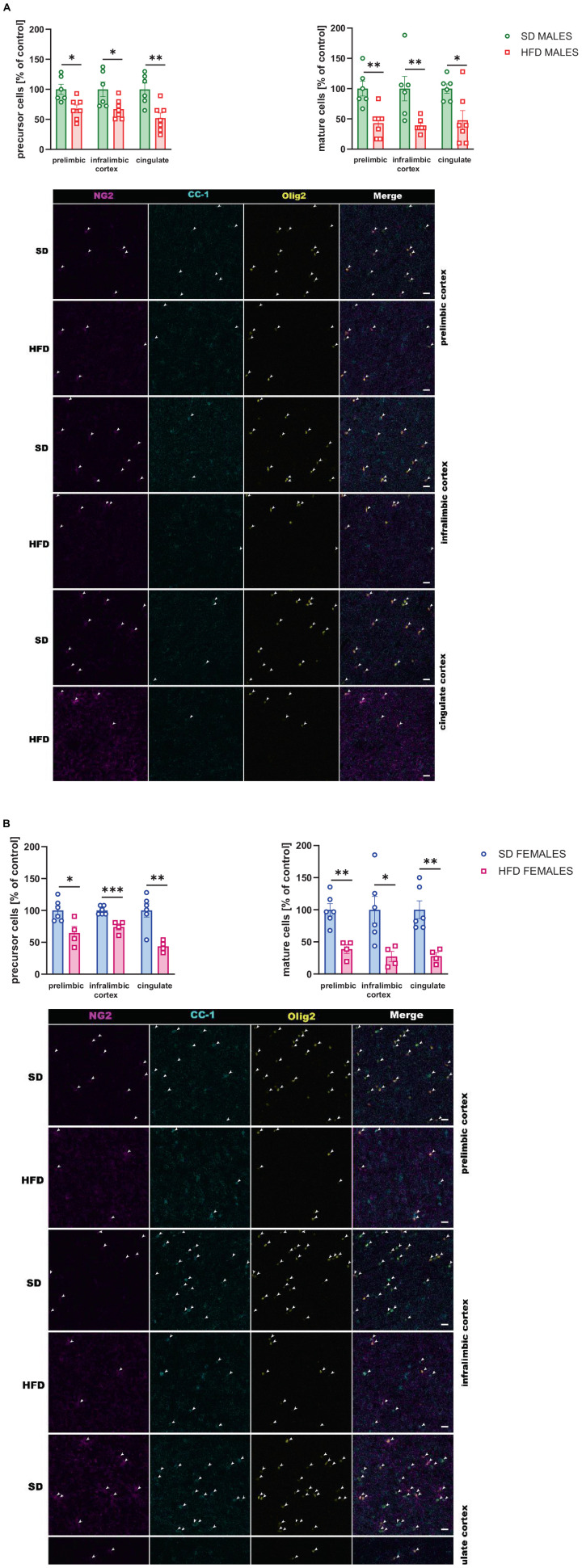
Effect of a maternal high-fat diet (HFD) during pregnancy and lactation on the number of oligodendrocyte precursor cells (NG2^+^/Olig2^+^ cells) and mature oligodendrocyte cells (CC-1^+^/Olig2^+^ cells) in the prelimbic, infralimbic, and cingulate cortex of adult **(A)** male and **(B)** female offspring. Representative images of channels showing NG2, CC-1, Olig2, and Merge immunofluorescence staining were shown. The scale bar represents 20 μm. Data are presented as the mean ± SEM. *N* = 5–7 rats/group. *p < 0.05, **p < 0.01, ***p < 0.001 vs. standard diet (SD).

The reduced levels of the number of oligodendrocyte precursor cells were observed in the prelimbic (*t* = 3.091, df = 11, *p* = 0.01), infralimbic (*t* = 2.559, df = 11, *p* = 0.027), and cingulate (*t* = 3.611, df = 11, *p* = 0.004) cortex of the adult male offspring following maternal HFD during pregnancy and lactation (Figure 7A). Similarly, the number of mature oligodendrocyte cells was reduced in the prelimbic (*t* = 3.882, df = 11, *p* = 0.003), infralimbic (*t* = 3.190, df = 11, *p* = 0.009), and cingulate (*t* = 2.779, df = 11, *p* = 0.018) in the adult male cortex (Figure 7A).

Moreover, in female adult offspring the reduced number of oligodendrocyte precursor and mature oligodendrocyte cells were observed in all structures examined following maternal HFD during pregnancy and lactation [*precursor cells*: prelimbic (*t* = 2.974, df = 8, *p* = 0.018), infralimbic (*t* = 5.103, df = 8, *p* = 0.001), cingulate (*t* = 4.193, df = 8, *p* = 0.003) cortex; *mature cells*: prelimbic (*t* = 4.669, df = 8, *p* = 0.002), infralimbic (*t* = 2.726, df = 8, *p* = 0.026), cingulate (*t* = 4.053, df = 8, *p* = 0.004) cortex] (Figure 7B).

## 4 Discussion

Exposure to a maternal HFD can induce alterations in the structure, function, and maturation of the central nervous system, potentially leading to the onset of neuropsychiatric disorders in the offspring. We observed an increased immobility time and a reduced swimming time in the forced swimming test with no changes in the locomotor activity in adolescent and adult offspring, which suggests a depressive-like phenotype in those animals. The latter changes are confirmed in our previous data, in which we showed that modifications in maternal nutritional intake during the gestational and lactational periods are pivotal to producing a depression-like behavior in offspring (Gawlinska et al., 2019, Gawlińska et al., 2020b; Gawliński et al., 2021), as well as dams fed HFD before breeding also provoked the same phenotype in offspring (Bayandor et al., 2019). Meanwhile, it was observed that adult female rats, but not their adolescent counterparts, exhibited a noteworthy decrease in their consumption of a sweet solution following maternal HFD exposure suggesting the possibility of long-lasting changes induced by maternal HFD exposure (Hassan et al., 2019; Gawlińska et al., 2020b). Given that biological sex is a predictive factor for the lifetime prevalence of depression—with higher rates in women than in men (Sikes-Keilp and Rubinow, 2021) - this reduced sucrose preference may indicate potential anhedonia in these adult rats, a commonly observed symptom of depression (Cooper et al., 2018). Notably, consumption patterns in adult male offspring also showed reduced preference for sucrose solution when exposed to a maternal HFD rich in saturated fatty acids (Gawlińska et al., 2020b), it seems that these behavioral discrepancies in depression-like phenotype could be influenced by the specific composition of the maternal diet. The mechanisms behind these sex-specific responses, potentially involving differential hormonal influences on brain development and function, warrant further investigation. The observed sex dimorphism emphasizes the importance of personalized approaches in the prevention and treatment of depression, acknowledging the complex interplay between biological sex, developmental stage, and environmental exposures.

Analysis of gene expression in the PFCTX of adolescent rats identified 11 top differentially expressed genes related to myelination affected by maternal diet. Interestingly, maternal HFD was not associated with alterations in the expression of these genes in the hippocampus, suggesting that myelin-related changes are region-specific and the PFCTX seems to be more sensitive to associations with changes in myelination induced by maternal diet. Myelin-related changes induced by a maternal HFD during pregnancy and lactation in different brain regions could influence the functions of those areas. In the case of depression, reduced myelination in the PFCTX may be associated with impairments in executive functions and emotional regulation, which are often observed in individuals with depression (Kaya and McCabe, 2019). On the other hand, the hippocampus is a brain region more closely linked to memory and emotional functions, rather than executive control as seen in the PFCTX. Therefore, changes in myelination seem to be more noticeable and significant in the PFCTX in the context of depression. Additionally, numerous developmental investigations focusing on depression have consistently revealed the presence of myelin abnormalities in the PFCTX (Smaga, 2022). Neuroimaging studies showed decreased myelin levels in the lateral PFCTX in depressed patients with a greater number of depressive episodes (Sacchet and Gotlib, 2017). Additionally, myelin-related alterations in the PFCTX were observed in animal models of depression induced by stress (Birey et al., 2015; Yang et al., 2016, 2017; Lehmann et al., 2017; Liu et al., 2018) or in olfactory bulbs lesions (Takahashi et al., 2021).

Decreased mRNA levels of the genes *Mog*, *Mal*, *Mag*, *Mobp*, *Cnp*, *Klk6*, *Cldn11*, *Gjb1*, and *Tf*, and increased mRNA levels of *Tfrc* were observed in male adolescent offspring following maternal HFD during pregnancy and lactation. These changes in the gene expression had functional implications, this was evident in the reduced protein levels of several proteins (MOG, MAL, MAG, MOBP, CNPase, kallikrein 6, claudine 11, GJB1, and transferrin) in the PFCTX. At the same time reduced expression levels of genes *Mog*, *Mal*, *Klk6*, *Gjb1*, and *Tf* and increased level of *Tfrc* were observed in female adolescent offspring, while their protein levels did not change in this structure. These reduced mRNA levels of *Mog*, *Mal*, *Cnp*, *Klk6*, and *Tf* in male and *Mog*, *Mal*, *Klk6*, and *Tf* in female offspring persisted even into adulthood. Conversely, elevated mRNA levels of *Tfrc* were maintained only in adult female offspring following maternal HFD during pregnancy and lactation. Furthermore, certain protein levels (specifically MOG, MAL, CNPase, kallikrein 6, and transferrin) remained altered even during adulthood in males. Interestingly, reduced levels of two proteins, MOG and kallikrein 6, were observed in adult female offspring. It seems that the reduced levels of these proteins observed in offspring may be associated with a depression-like phenotype following maternal HFD during pregnancy and lactation in offspring. These proteins collectively orchestrate integral roles in the process of myelination and contribute significantly to the optimal functioning and maintenance of the myelin sheath. Postmortem studies on the brains of major depressive disorder patients confirmed less intense myelin staining in the cortical regions (Tham et al., 2011), as well as this disorder leads to a reduced oligodendroglial-cell number in various brain regions including the PFCTX (Hayashi et al., 2011). Similarly, the downregulation of genes related to oligodendrocyte function and components of myelin sheaths including *CNP, MAG, MAL, MOG*, and *MOBP* were observed in depressive patients (Aston et al., 2005; Sibille et al., 2009). Reduction of these genes was also observed in our preclinical study, which may suggest a potential correlational role in the maternal diet-induced depressive-like phenotype in offspring.

The reduction in myelin-related genes and proteins in the PFCTX of offspring following maternal HFD exposure might be related to the impact of the diet on overall metabolic health and subsequent neurodevelopmental processes. Maternal HFD exposure has been associated with metabolic disorders such as obesity and diabetes, which can lead to systemic inflammation (Harmancıoğlu and Kabaran, 2023). This inflammation could potentially be associated with the CNS and disrupt normal myelination processes (Graf et al., 2016). MOG, primarily localized in the outermost layer of the myelin sheath, contributes to myelin stabilization and interactions with the immune system (Tanaka et al., 2023). Despite the primary role of MOG being associated with myelin and its interaction with the immune system, there exists a paucity of research specifically investigating the direct correlation between MOG and depression. Autoimmune responses against MOG have been associated with demyelinating disorders such as multiple sclerosis (Mader et al., 2023). It has been shown a correlation between this disorder and an augmented risk of depression (Jacobs et al., 2023), while depression manifests as prevalent comorbidity in individuals exerting a significant impact on their overall quality of life (Feinstein et al., 2014). It is imperative to note that the mRNA levels of *Mog* were diminished in the PFCTX in both male and female adolescent offspring following maternal HFD exposure, and this reduction persisted into adulthood. Furthermore, we also observed a parallel decrease in the functional (protein) levels of MOG. These molecular changes are correlated with changes in behavior (increased immobility time) and may play a critical role in the development of depression-like behavior observed in the offspring as a result of maternal HFD exposure. Analogously, reduced *Mog* mRNA levels were observed in mice subjected to social isolation (Liu et al., 2012) and chronic social defeat stress (Lehmann et al., 2017), suggesting a potential role of this molecule in the depression-like behavior in offspring observed in our study.

Interestingly, the reduction in CNPase (mRNA and protein) levels was observed only in male adolescent and adult offspring. Our results delineate sex-specific divergences in the modulation of myelin-related gene expression as a consequence of maternal and early-life dietary influences. These observations are congruent with existing literature positing that epigenetic mechanisms operative within the CNS display sexual dimorphism. Such differences are likely attributable to the variable expression of estrogen receptors, the influence of neuroinflammatory mediators, differential exposure to hormones during critical developmental windows, and inherent cellular disparities rooted in chromosomal sex (McCarthy and Arnold, 2011). CNPase is required for the maintenance of axon-glia interactions at nodes of Ranvier (Rasband et al., 2005). Other proteins involved in axon-glial interactions, MAG and MOBP, which also play a pivotal role in myelin stabilization, were reduced (mRNA and protein levels) only in adolescent male offspring following maternal HFD. Additionally, decreased *Mal* and *Tf* mRNA levels were observed in all offspring, but their protein levels were reduced only in male offspring following maternal HFD. MAL is a protein involved in the transport of lipids and myelin proteins to the growing myelin sheath, while transferrin, an iron transport protein, is key in the formation of myelin (Todorich et al., 2009). These proteins play important roles in maintaining the function of myelin and any disruption in the myelination may be associated with behavioral deficits in offspring, whose mothers were fed HFD during pregnancy and lactation. Decreased levels of MAG, MAL, CNPase, and MOBP, were also confirmed in animal models of depression (Liu et al., 2012, 2018; Makinodan et al., 2012; Luo et al., 2019; Takahashi et al., 2021) and in the human postmortem study in depressed patients (Aston et al., 2005; Rajkowska et al., 2015). Moreover, the reduced levels of claudin 11 and Gjb1 (mRNA and protein) were observed only in adolescent male offspring, whose mothers were fed HFD during pregnancy and lactation. GJB1 is a gap junction protein facilitating direct intercellular communication among neighboring cells, whereas claudin 11 is a tight junction protein integral to the creation and upkeep of myelin sheaths. Claudin 11 achieves this by establishing tight junctions between neighboring myelin-forming cells. Taken together an adolescent male brain appears to be more susceptible to environmental factors including a maternal HFD during pregnancy and lactation, which was also observed in our previous study (Gawliński et al., 2021).

In the present study, diminished levels of kallikrein 6 were observed following maternal HFD exposure in adolescent male (both mRNA and protein levels) and female (mRNA levels) offspring. This reduction was enduring, persisting into adulthood in both male (mRNA and protein levels) and female (mRNA and protein levels) offspring. Kallikrein 6, synthesized by mature oligodendrocytes within the CNS, may contribute to alterations in the myelin milieu, acting as a significant regulator of myelin proteins (Bando et al., 2006). Interestingly, reduced *KLK6* gene expression was also observed in the PFCTX of patients diagnosed with depression (Boda, 2019), which may suggest a potential correlative role in this disorder. These observations align with the hypothesis that kallikrein 6 could be involved in the depressive-like behavior observed in offspring following maternal HFD exposure during pregnancy and lactation.

The altered mRNA expression levels of *Tf* and *Tfrc* in offspring following maternal HFD exposure suggest a disruption in iron metabolism within the PFCTX (Cheli et al., 2020). Transferrin, the main protein responsible for iron transport in the brain, may have reduced expression reflecting a state of altered iron homeostasis. This could potentially affect myelination and synaptic plasticity—processes that are dependent on iron (Beard and Connor, 2003; Markova et al., 2019). Conversely, the upregulation of *Tfrc* mRNA levels may indicate an adaptive response to maintain iron uptake in light of reduced *Tf* availability. In females, the persistence of these alterations into adulthood underscores a sustained impact of early-life HFD on iron regulatory mechanisms. This could underlie the enduring changes in myelin protein expression and the associated depression-like behaviors. For males, the continued reduction of transferrin at both the mRNA and protein levels into adulthood could suggest a sex-specific regulatory mechanism. This may be indicative of a compensatory response to altered iron dynamics resulting from maternal HFD, or it may reflect inherent sex-based differences in iron utilization for neural processes. These findings underscore the critical role of iron in neural development and how maternal diet can impact the availability of this trace element, with significant implications for offspring brain function and behavior. Future research should aim to delineate the precise pathways through which maternal HFD disrupts iron metabolism and the extent to which these changes contribute to the pathophysiology of depression-like phenotypes.

The myelin-related changes in genes and proteins observed in the PFCTX of offspring following maternal HFD were also associated with a lower number of immature oligodendrocyte cells in the prelimbic and cingulate cortex and with a lower number of mature oligodendrocyte cells in the infralimbic cortex of female offspring. In contrast, in male offspring, an increase in the number of mature oligodendrocytes was observed in the cingulate cortex. Alterations in cell counts were notably pronounced among older animals. In fact, the number of oligodendrocyte precursor cells and mature oligodendrocytes reduced following maternal HFD exposure in adult offspring in almost all brain regions examined. Previous studies reported a higher number of immature oligodendrocyte cells and a lower number of mature oligodendrocytes in the PFC in mice following social defeat stress (Lehmann et al., 2017; Bonnefil et al., 2019), maternal separation (Yang et al., 2017), and olfactory bulbectomized mice (Takahashi et al., 2021). Thus, it may be postulated that maternal diet-induced alterations in myelination may be associated with the reduction of immature and mature oligodendrocytes in the PFCTX in later life. Interestingly, in a related study, mice whose mothers were fed an HFD pre-pregnancy, during pregnancy, and lactation did not exhibit changes in the levels of mature oligodendrocytes in the corpus callosum, whereas increased myelin density was associated with a reduced area of cytosolic myelin channels in male compared to female offspring, alongside microglia-dependent alterations (Bordeleau et al., 2021). Maternal HFD has been implicated in the dysregulation of neurodevelopmental processes, notably through the activation of astrocytes and microglia, leading to the promotion of neuroinflammation. This environment can potentially lead to the concurrent activation of astrocytes and microglia, instigating a cascade of neuroinflammatory responses that disrupt myelination and synaptic development in offspring (Bordeleau et al., 2020, 2022). Activation of microglia, the brain’s resident immune cells, can further amplify neuroinflammatory signaling and affect neurodevelopmental processes by altering cytokine profiles and affecting the phagocytic clearance of synaptic elements. These glial-mediated changes are significant as they can alter neural connectivity and function, which are critical during brain development, potentially leading to varied behavioral outcomes in offspring. Such early-life inflammatory exposures are associated with cognitive deficits and altered affective behaviors, which may present sex-based differences due to distinct hormonal and immune system interactions. A comprehensive understanding of the impact of maternal HFD on both astrocyte and microglia activity and the subsequent neuroinflammation is thus crucial for developing targeted interventions to mitigate potential adverse effects on offspring brain health.

In summary, the present findings indicate of several myelin-related changes in brain offspring followed by maternal HFD. The transcriptional downregulation of genes linked to oligodendrocyte development and structural components of myelin, associated with maternal HFD exposure, could suggest that one of the diet-induced effects is associated with abnormal myelination in the offspring’s brain. The depressive-like phenotype observed in offspring may be correlated with dysregulation of several genes and proteins in the PFCTX, especially of MOG, MAL, CNPase, kallikrein 6, and transferrin in male offspring, as well as of MOG and kallikrein 6 in female offspring, which persist even into adulthood. Maternal HFD was also associated with long-lasting changes, manifested by the reduction of immature and mature oligodendrocytes in the PFCTX in adult offspring. Alterations in their expression, function, or interactions may impact myelin function potentially leading to various psychiatric disorders including depression. However, the precise mechanisms linking maternal HFD, myelination, and depression in offspring are complex and not fully understood. Further research is needed to elucidate these relationships. Nonetheless, our study provides important insights into the potential involvement of several proteins in the etiology of depression-like behavior, particularly in the context of maternal HFD exposure.

## Data availability statement

The original contributions presented in the study are publicly available. This data of RNA sequencing can be found here: https://github.com/ippas/ifpan-smaga-diet and https://www.ncbi.nlm.nih.gov/sra/PRJNA977708. Further inquiries can be directed to the corresponding author.

## Ethics statement

The animal study was approved by the Local Ethics Commission at the Maj Institute of Pharmacology Polish Academy of Sciences, Kraków, Poland (255/2021, 26 August 2021). The study was conducted in accordance with the local legislation and institutional requirements.

## Author contributions

MFr: Data curation, Formal analysis, Methodology, Validation, Writing—review and editing. PS: Data curation, Formal analysis, Methodology, Validation, Writing—review and editing. KG: Data curation, Formal analysis, Methodology, Validation, Writing—review and editing. MB: Data curation, Methodology, Validation, Visualization, Writing—review and editing. MK: Data curation, Visualization, Writing—review and editing. MF: Writing—review and editing. IS: Conceptualization, Data curation, Formal analysis, Funding acquisition, Investigation, Methodology, Project administration, Validation, Visualization, Writing—original draft, Writing—review and editing.

## Footnotes

1
https://intelliseq.com

2
https://github.com/ippas/ifpan-smaga-diet
